# Does Self-Care Make You a Better Leader? A Multisource Study Linking Leader Self-Care to Health-Oriented Leadership, Employee Self-Care, and Health

**DOI:** 10.3390/ijerph19116733

**Published:** 2022-05-31

**Authors:** Katharina Klug, Jörg Felfe, Annika Krick

**Affiliations:** 1 Faculty of Business Studies and Economics, University of Bremen, 28359 Bremen, Germany; 2 Department of Work, Organizational and Business Psychology, Helmut Schmidt University Hamburg, 22043 Hamburg, Germany; felfe@hsu-hh.de (J.F.); krick@hsu-hh.de (A.K.)

**Keywords:** health-oriented leadership, self-care, employee health, leader well-being, leadership, multilevel analysis

## Abstract

Leadership plays an important role in employee well-being. In light of a growing research interest in leaders’ resources as determinants of healthy leadership, it is not yet clear how leaders’ behavior regarding their own health (self-care) may trickle down to employees. Drawing on Conservation of Resources Theory and the model of Health-Oriented Leadership, this study tests two mechanisms through which employees may benefit from self-caring leaders: (a) through staff care, that is, concern for their employees’ health (improved leadership hypothesis); and (b) through a direct relationship between leaders’ and employees’ self-care (role-modeling hypothesis). In turn, both staff care and employee self-care would relate positively to employee health. Multilevel path models based on a sample of *N =* 46 supervisors and 437 employees revealed that leader self-care was positively related to leader-rated staff care at Level 2, which was positively related to employee-rated staff care at Level 1. In turn, employee-rated staff care was positively related to employee health. The findings support the improved leadership hypothesis and underline the importance of leader self-care as a determinant of healthy leadership.

## 1. Introduction

Leaders can affect their employees’ health in various ways, for example, via social support [1], influencing work design [2], crossover of stress [3], or role-modeling health behavior [4]. At the same time, managing people is a demanding task and leaders themselves may experience stress [5,6]. Their stress may impact their behavior toward employees. Recent research has shown an increasing interest in leaders’ own resources and well-being as influencing factors of leadership [7,8,9]. In this context, leader self-care in terms of personal efforts to manage one’s health at work [10] may play an important role in two ways: by improving their leadership behavior or through a direct role-modeling effect on employee self-care.

The aim of this study was to test which of these two mechanisms is more likely. Using the model of Health-oriented Leadership (HoL) [10], we investigated how leader self-care relates to healthy leadership (staff care), employee self-care, and employee health. The HoL model differentiates *staff care*, comprising leaders’ attitudes and behavior toward employee health, from leaders’ and employees’ *self-care* in terms of attitudes and behavior toward their own health. The model suggests that staff care protects and improves employees’ resources, for example, by improving working conditions; see also [10,11]. Staff care also facilitates and encourages employee self-care, which in turn relates to improved well-being [12,13].

Leader self-care has been proposed as “the foundation of health-promoting leadership” [10], yet this assumption needs to be empirically tested, including the mechanisms why this should be the case. Whereas there is a lot of evidence for the positive effects of staff care and employee self-care, e.g., [14,15,16], it remains unclear whether leader self-care trickles down and how employees may benefit from self-caring leaders. Two different mechanisms may link leader self-care to employee outcomes: first, leaders who value and protect their own health may also be motivated to do so for others [9,17], which would be recognized by employees and, in turn, relate positively to their health. Second, the HoL model considers leaders as potential role models for employees, which suggests that employees of self-caring leaders would also report higher self-care and, in turn, better health. Based on these considerations, we argue for the theoretical and practical relevance of leader self-care as an important part of healthy leadership.

We used a multi-source design and multilevel path models to test these mechanisms and investigate the direct and indirect relationships between leader self-care, staff care, employee self-care, and employee health. We focused on irritation, psychosomatic complaints, and overall health to capture a range of proximal, work-specific, and more distal and general health outcomes. Irritation and psychosomatic complaints can be seen as capturing immediate cognitive-emotional strain reactions versus more long-term physical reactions to work stress [18], respectively, whereas self-rated health provides a measure of people’s overall health status beyond the work domain [19,20]. The main theoretical basis of the study is COR theory [21], but we also draw on Social Learning Theory [22] to understand leaders as role models. The study contributes to the literature in several ways. First, we test different mechanisms of why leader self-care may relate to employee outcomes by simultaneously considering indirect relationships via improved leadership and role-modeling. Second, we add to the growing research field on leader resources and wellbeing as determinants of healthy leadership [8,9] by demonstrating the relevance of leader self-care. Methodologically, we extend previous single source studies by integrating leaders’ and employees’ perspectives on both self-care and staff care. From a practical perspective, leader self-care may be an important topic for occupational health management: if leaders are expected to promote other people’s health at work, they need to be equipped with the skills and capacities to do so for themselves. 

### 1.1. The Model of Health-Oriented Leadership

The determination of what constitutes “healthy” leadership remains a challenge in the literature [5]. On the one hand, a large number of studies have linked constructive and destructive leadership to employee well-being [23,24,25]. On the other hand, because established leadership concepts traditionally focus on performance, domain-specific concepts have been introduced to explicitly capture health-relevant attitudes and behaviors; see [26] for a critical review. Actually, health-specific leadership has been shown to explain variance in health above and beyond general concepts such as transformational leadership or leader–member exchange [10,15,27,28]. 

This study is based on the model of Health-oriented Leadership [HoL; 10], which conceptualizes how leaders treat employee health, but also how leaders and employees each treat their own health at work (self-care). The model includes three dimensions: (1) *staff care* denotes employee-directed, health-specific leadership; (2) *leader self-care* and (3) *employee self-care* each denote leaders’ and employees’ attitudes and behavior regarding their own health at work. Staff care and self-care each consist of three facets: (1) *Value* describes the value leaders and employees ascribe to health, that is, the extent to which they show interest in and prioritize health at work, including the extent to which leaders and employees feel responsible for their employees’ and their own health, respectively. (2) *Awareness* describes perceptiveness regarding employees’ or one’s own well-being and stress, which is reflected in knowing one’s own and employees’ limits with regard to stress and strain, and noticing health-related warning signals. (3) *Behavior* refers to health-relevant behaviors at work aiming to reduce stress and protect health, ranging from taking breaks, improving employees’ or one’s own work organization, to (encouraging) participation in workplace health promotion programs [10]. In this study, we focused on the behavioral facets of staff care and self-care, which have been shown to relate more strongly to employee health outcomes [29].

Regarding self-care, there are different definitions in the literature, and facets subsumed under the label self-care range from physical activity, sleep and eating habits, seeking counseling or self-medication, to spiritual aspects and practices such as meditation [30,31,32]. Self-care has also been linked to self-compassion and mindfulness [31,33,34]. Broadly speaking, self-care can be defined as people’s personal activities to protect and improve their own health and well-being. Self-care is self-initiated and non-professional, thus reflecting everyday health-related attitudes and behavioral routines [32]. In the HoL model, self-care is the self-directed equivalent to staff care, that is, self-care includes leaders’ and employees’ values, awareness, and behavior regarding their own health. The behavioral component of self-care, the focus in this study, encompasses self-guided activities and routines aimed at protecting and fostering well-being at work, e.g., taking enough time to recover, seeking support, and avoiding overtime [10].

Several studies support the validity of the HoL framework. Franke et al. [10] provided initial evidence of the construct and incremental validity of the HoL scales, and linked employee perceptions of both self-care and staff care to irritation, psychosomatic complaints, overall health, and work–family conflict. Others have since then replicated the scale structure and incremental validity beyond transformational leadership [13,15], in addition to relationships with irritation, psychosomatic complaints, and overall health [16,35,36,37,38]. Self-care and staff care have also been linked to work engagement [15,17], exhaustion and burnout [12,13,15,17,39], physical health [13,14], different indicators of mental health [13,14,29], subjective well-being [13], and performance [35]. Empirical evidence also supports the notion that self-care and staff care function as resources. Staff care has, for example, been shown to buffer the effects of job demands on strain, health, and job satisfaction [38], and to facilitate employees’ participation in workplace health promotion programs [40], while baseline levels of self-care have been shown to predict a stronger increase in heart rate variability during a mindfulness intervention [41]. Several studies also support the mediating role of self-care [10,12,13,15], suggesting that the positive health effects of staff care can be partly attributed to facilitating employee self-care. As most studies focused on employee perceptions, the role of leader self-care is, as yet, underexplored; see [17,29] for exceptions. Below, we elaborate why leader self-care may be an important determinant of healthy leadership, either by improving staff care or by trickling down directly to employee self-care through role-modeling.

### 1.2. Linking Leader Self-Care to Employee Perceptions and Behavior

The idea that leader self-care is the foundation of health-oriented leadership [10] rests on two assumptions. First, the HoL model relates leader self-care to staff care, such that self-caring leaders would be more willing and able to provide staff care, which in turn enables employee health (the improved leadership hypothesis). Second, the model considers leaders as role models for their employees, such that self-caring leaders inspire employees to engage in self-care (the role-modeling hypothesis). In the following, we review theoretical arguments and evidence for both mechanisms to develop our hypotheses.

#### 1.2.1. The Improved Leadership Hypothesis

The first avenue through which employees may benefit from self-caring leaders is improved staff care. A leader’s work environment is usually characterized by a high workload, cognitive complexity, and multitasking, plus a high degree of responsibility, both for results and for others’ performance and well-being [6,42,43]. Self-care can help leaders to manage these demands, reduce stress, and protect their resources. Self-care can therefore free up capacities to create resources for others, such that leaders are better able to pay attention to their employees’ health, and identify and seize opportunities to reduce stress and provide support [8,9]. In a similar vein, theoretical [44,45] and empirical [46] works on the relation between leadership and self-leadership suggest that self-leadership is a precondition of effectively leading others. Finally, self-caring leaders may also be more motivated to show staff care toward their employees out of a desire for consistency or authenticity between what they value for themselves and how they behave toward others [47]. In turn, leaders who neglect their own health would be less willing to invest much effort in their employees’ health. 

Supporting these considerations, leaders’ workload has been shown to correlate with a reduced capacity to provide support to employees [48], and meta-analyses show positive correlations between leader well-being and constructive leadership [7,24]. Because we expect the positive effects of leader self-care to unfold through leader resources and motivation, we first focus on leaders’ own perspective on their staff care [17]. We expect that self-caring leaders will feel more able and willing to improve their employees’ well-being than those with low self-care, and consequently report higher levels of staff care:

**Hypothesis** **1:**  *Leader self-care is positively related to leader-rated staff care*.

Furthermore, we expect that leaders’ efforts to engage in staff care will be noticed by employees and reflected in employee-rated staff care, resulting in a considerable self-other agreement. First and foremost, leader and employee ratings of staff care refer to the same entity (i.e., leader’s attitudes and behavior toward employees), and thus share a common core. Second, leaders who view themselves as caring for their employees’ well-being will express this through behavior and communication, which informs employees’ judgements of staff care (e.g., talking to employees about ways to reduce stress, [10]). Accordingly, the literature typically reports a moderate self-other agreement in ratings of leadership behavior [29,49,50]. By linking leader and employee ratings of staff care, we can validate both perspectives and rule out that employee perceptions are merely the result of subjective attribution processes, or that leaders’ self-ratings are merely the result of positive self-evaluations and social desirability [51]. Although investigating agreement as such is beyond the scope of this paper, we expect leader and employee perceptions of staff care to correlate:

**Hypothesis** **2:**  *Leader-rated staff care is positively related to employee-rated staff care*.

Generally, employee perceptions of positive leadership behaviors relate to employee health [23]. As a positive leadership behavior, staff care represents a resource for employees that should reduce stress and improve well-being. Accordingly, previous research has linked employee-rated staff care to various indicators of physical and mental health [12,13,14,15,16,35]:

**Hypothesis** **3:**  *Employee-rated staff care is positively related to employee health*.

From Hypotheses 1–3, it follows that leader self-care is indirectly linked to employee health via leader-rated staff care and employee-rated staff care. Although we expect a positive relationship between leader self-care and staff care, previous research with employee ratings suggests that this is not always consistent and not all leaders will translate their own self-care into staff care: Some may, for example, show concern for their employees’ health while neglecting their own, whereas others may engage in self-care while not feeling responsible for their employees’ health [16]. In turn, leaders’ and employees’ ratings of staff care will not agree perfectly either [29,49,50]. For the improved leadership hypothesis, we therefore consider both perspectives and expect employees to report better health to the extent that self-caring leaders show more staff care and employees also observe more staff care. From Hypotheses 1–3 follows:

**Hypothesis** **4:**  *Leader self-care is indirectly associated with employee health via leader-rated staff care and employee-rated staff care*.

#### 1.2.2. The Role-Modeling Hypothesis

Social Learning Theory [22] explains why employees may adopt behavior from their leaders. Supervisors serve as role models due to their influential position, as they set standards of what is accepted and desirable behavior at work [47,52]. Applying Social Learning Theory to self-care, leaders can be salient role models by demonstrating self-caring behaviors, such as taking regular breaks, participating in health activities, or effective time and stress management. Employees may also perceive self-care as rewarding when they observe positive effects on their leader’s well-being. Finally, showing that they value health, self-caring leaders can elicit positive expectancies among employees who would anticipate being rewarded or at least not punished for engaging in self-care. These aspects encourage employees to emulate their leader’s self-care through vicarious learning [22]. In contrast, leaders who engage in health-risking behaviors and overwork themselves communicate to employees, if only implicitly, that they need to prioritize performance over health to be successful in the organization. In consequence, employees are discouraged from self-care, because they may perceive it as not being feasible or may even fear negative repercussions. 

There is ample evidence that behavior trickles down the organizational hierarchy in this way [53]. However, studies in the occupational health domain tend to address the direct crossover of strain between leaders and employees more often than health specific behavior (e.g., [54,55]). Studies showing positive relationships between leaders’ and employees’ absenteeism and presenteeism provide empirical support for a potential link between leaders’ and employees’ self-care [4,56]. The role-modeling mechanism suggests a direct relationship between leader self-care and employee self-care. In turn, as employee self-care relates to improved well-being [10,12,13], leader self-care would indirectly relate to employee health via employee self-care. We expect that, when leaders show higher self-care, their employees show higher self-care and also report better health:

**Hypothesis** **5:**  *Leader self-care is positively related to employee self-care*.

**Hypothesis** **6:**  *Employee self-care is positively related to employee health*.

**Hypothesis** **7:**  *Leader self-care is indirectly related to employee health via employee self-care*.

## 2. Materials and Methods

### 2.1. Sample and Procedure

The sample consisted of 466 administrative employees matched to their 47 immediate supervisors from a German health insurance provider. The leaders volunteered to participate in the study as part of a leadership program and all leaders who signed up for the program provided data. The leaders were contacted first, then employees completed the survey during the following 2–3 weeks. Response rates on the team level ranged from 29 to 100%, with an average of 75%. In collaboration with the organization’s HR department, participants were informed about the study, including the voluntary nature of participation and confidential treatment of the data, and given the opportunity to clarify remaining questions with the researchers. Respondents gave their informed consent and completed an online survey during their working time. 

The majority of the employees were women (78%) and, on average, were 45 years old (*M* = 44.67, *SD* = 10.94). Employees had worked with their leaders between one and 27 years (*M* = 4.35, *SD* = 4.37). The majority (70%) of the leaders were also women and, on average, were about 47 years old (*M* = 46.98, *SD =* 8.26). The leaders reported a span of control ranging from 5 to 29 employees (*M* = 13.89, *SD =* 5.92). Around two-thirds (60%) of the leaders were team leaders, whereas others were in higher positions (32% division managers, 8% executive board members). The observed team size per leader ranged from 5 to 19 employees, with an average of about 11 per team (*M* = 11.29, *SD* = 4.03).

### 2.2. Measures

Both employees and leaders completed a questionnaire on health-oriented leadership, strain, and health. Unless stated otherwise, all items were rated on a 5-point scale ranging from 1 = not at all true to 5 = completely true. Self-care and staff care were each assessed with the Health-oriented Leadership Questionnaire [57]. Self-care and staff care each contain sets of overlapping but not completely identical items that differ in their perspectives and targets of behavior (self-directed versus employee-directed, rated by leaders or employees, respectively).

*Self-care.* Leaders and employees each rated their own self-care, measured with 14 items assessing health behavior (e.g., “I make sure to have enough time for recovery”). Reliability for the overall scale was α = 0.85 among leaders and α = 0.79 among employees.

*Staff care.* Employee-directed leadership was measured with the staff care behavior scale. Leaders completed the self-report version and rated their own staff care with 19 items (e.g., “I make sure my employees have enough time for recovery”). Employees completed the employee version of the scale and rated their supervisors’ staff care behavior with 19 items (e.g., “My supervisor makes sure there is enough time for recovery”). Reliability for the scales was α = 0.85 among leaders and α = 0.93 among employees.

*Health indicators.* We investigated irritation, psychosomatic complaints, and employees’ general health status as commonly used indicators to capture both work-related strain and also as a potential spill-over to health outside work [18]. Employees reported their health in terms of cognitive-emotional strain, psychosomatic complaints, and their overall health status. Strain was measured with the irritation scale [58], consisting of eight items (e.g., “I get grumpy when others approach me”; α = 0.86 among both leaders and employees). The scale allows the measurement of irritation both with two subscales and as a global index [59]. We used the global index to capture employees’ overall strain (for descriptive purposes, we also report descriptive statistics and bivariate correlations in Table 1 for cognitive and emotional irritation, respectively). Psychosomatic complaints were measured with five items from the scale by Mohr [60] (e.g., “I often suffer from headaches, tensions or back problems”; α = 0.70 among leaders and α = 0.65 among employees). The overall health status was measured with a single item from the German COPSOQ questionnaire [61], asking participants to rate their current health status on an 11-point scale from 0 = worst conceivable health to 10 = best conceivable health. 

*Control variables.* We adjusted the analyses for a number of factors that may affect the relationships between leadership and health indicators. Namely, we controlled for employee age and gender, because they relate to strain and health [62,63]. Because employees and leaders develop a mutual understanding over time that may affect their interpretation and agreement with regard to staff care, we controlled for employees’ tenure with the leader [64]. We also controlled for the leaders’ span of control, which may affect the effectiveness of their staff care [65]. We adjusted our analyses for relationships of control variables with employee health, but also with employee self-care and staff care as mediator variables. 

We conducted a confirmatory factor analysis (CFA) on the HoL scales to test their structure and reliability in the employee sample (a multilevel CFA would have required a larger leader sample given the number of items). Our hypothesized model with two separate factors (self-care and staff care) showed a satisfactory and better fit compared to a one-factor model (one factor: *χ^2^*(481) = 1560.94, *p* < 0.001; CFI = 0.80, RMSEA = 0.069, SRMR = 0.081; two factors: *χ2*(480) = 1155.71, *p* < 0.001, CFI = 0.88, RMSEA = 0.055, SRMR = 0.065, ∆*χ^2^*(1) = 106.36, *p* < 0.001). Standardized factor loadings for self-care ranged from 0.14 (“I participate in workplace health promotion programs”) to 0.72 (“After longer periods of stress, I make sure that things calm down again”), with an average of M = 0.46. Standardized factor loadings for staff care ranged from 0.24 (“From time to time we have to skip breaks when there is a lot to do in our team”) to 0.80 (“My leader makes sure not to neglect health topics”), with an average of M = 0.63 (see Table S1 in the Supplementary Materials). Moderate factor loadings were theoretically justified, given that the items for self-care and staff care each display a range of different behaviors related to health at work. Considering the good internal consistencies (*α* = 0.79 for self-care; *α* = 0.93 for staff care) and previous studies validating the structure of the HoL constructs [10,13,15], we retained all items in the analysis to measure self-care and staff care behavior. 

Table 1 provides descriptive statistics and bivariate correlations for all study variables on the leader and employee level, respectively. 

### 2.3. Analyses

We calculated multilevel path models with Mplus 8 [66] using maximum likelihood with robust standard errors (MLR), to account for the nested structure of our data and test all hypotheses in one model. Whereas leader self-care and leader-rated staff care are, by definition, Level 2 variables, employee ratings were treated as Level 1 variables. Intra-class coefficients (ICCs) for employee variables in Table 1 indicate that small to medium proportions of the variance (between 5% for irritation and 20% for staff care) were attributable to the team level. Univariate ANOVAs indicated that team membership explained a significant proportion of the variance in employee irritation (F (46, 419) = 1.55, *p* = 0.015), psychosomatic complaints (F (46, 419) = 2.17, *p* < 0.001), and overall health (F (46, 411) = 2.03, *p* < 0.001), in addition to employee self-care (F (46, 418) = 1.75, *p* < 0.01) and staff care (F (46, 417) = 3.62, *p* < 0.001). 

**Figure 1:**
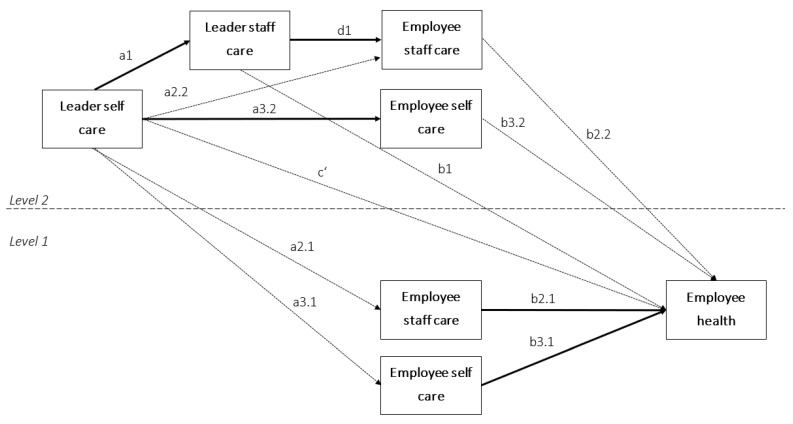
Conceptual model of the hypothesized mediation models from leader self-care (*X_j_* for a given team) to employee health (*Y*_2*ij*_) (a) via leader staff care (*M*_1*j*_ for a given team) and employee staff care ($M_{2j}^{-}$ for a given team and *M*_2*ij*_ for a given employee; within-team indirect effect: a1*d1*b2.1), and (b) via employee self-care ($M_{3j}^{-}$ for a given team and *M*_3*ij*_ for a given employee; indirect effect: a3.2*b3.1). The paths a2.1 and a2.2, and a3.1 and a3.2, are assumed to be equal; paths relevant to the hypotheses are in boldface.

To test Hypotheses 4 and 7, we calculated a cross-cluster level mediation model. There is some controversy as to how cross-level indirect effects in multilevel regression should be tested [67,68]. Following Pituch and Stapleton [67], we modeled relationships between Level 2 variables (e.g., leader self-care) and Level 1 mediators (e.g., employee-rated staff care) as individual rather than group-level relationships, because the theoretical focus of this study was on the individual level. Accordingly, we were interested in absolute effects, not contextual effects of employees’ relative position within their teams. In this case, it is more appropriate to specify indirect relationships at the individual rather than the group level [67]. For the same reason, we grand mean-centered all variables and controlled for between-team variance in employee ratings of self-care and staff care by including their respective aggregates on Level 2. This way, the cross-cluster mediation model allows differentiation of the between-team contextual indirect effects from the within-team indirect effects, the latter being relevant for our hypotheses [67]. We calculated our models combining the code provided by Stride et al. [69] to calculate Hayes’ [70] process models in Mplus and the code provided in Dietz et al. [4] to implement the cross-cluster level mediation [67]. The Mplus code is provided in the Supplementary Materials (Table S2). Figure 1 illustrates the model; the a-paths are assumed to be equal on both levels and thus only specified once, then multiplied with the respective within-team and between-team b-paths to obtain the respective indirect effect on each level. The total effects are then calculated as the sum of indirect effects on both levels and the direct effect (c’-path). To test our hypotheses, we first specified an indirect effect of leader self-care on employee health via leader-rated staff care and employee-rated staff care, respectively, (H4) as a 2-2-1-1 serial mediation. The relevant indirect effect is calculated from the paths from leader self-care to leader staff care (a1), from leader staff care to employee staff care (d1), to employee health (b2.1 and b2.2, respectively), while controlling for the specific indirect effects of each mediator (a1*b1; a2.2*b2.1 and a2.2*b2.2, respectively). The total effect is then calculated as the sum of the direct effect of leader self-care (c’) and all specific indirect effects, following Hayes [70]. Second, we specified indirect effects of leader self-care on employee health via employee self-care (H7) as a 2-1-1 mediation (a3.2*b3.1 on Level 1; a3.2*b3.2 on Level 2). 

## 3. Results

Table 2 shows the results of the path models for irritation, psychosomatic complaints, and overall health. The variables explained between 23% (psychosomatic complaints) and 31% of variance (irritation) in the health indicators on the employee level.

Hypotheses 1–4 addressed the improved leadership hypothesis. Specifically, Hypotheses 1 and 2 stated that leader self-care would relate to leader staff care, and that leader staff care would relate to employee staff care. The relationship between leader self-care and leader staff care was positive (*γ* = 0.62, *p* < 0.001), in addition to that between leader staff care and employee staff care (*γ* = 0.47, *p* < 0.01), after accounting for gender, age, tenure with the leader, and span of control. Hypotheses 1 and 2 were supported. In line with Hypothesis 3, employee staff care was negatively related to irritation (*γ* = −0.18, *p* < 0.01) and psychosomatic complaints (*γ* = −0.15, *p* < 0.05), and positively related to overall health (*γ* = 0.18, *p* < 0.01). Table 3 shows the indirect effects for the mediation hypotheses. The indirect effect of leader self-care on employee health via leader staff care and employee-rated staff care (H4) was significant for irritation (*γ* = −0.04, SE = 0.02, *p* < 0.05; 95%-CI [−0.08, −0.01]) and overall health (*γ* = 0.08, SE = 0.04, *p* < 0.05; 95%-CI [0.02, 0.15]), but missed the threshold for psychosomatic complaints (*γ* = −0.03, SE = 0.02, *p* < 0.10; 95%-CI [−0.07, −0.002]). Hypothesis 4 was partially supported. 

Hypotheses 5 and 6 postulated a direct positive relationship between leader self-care and employee self-care, and between employee self-care and health, respectively. The path from leader self-care to employee self-care was not significant (*γ* = 0.11, *p* = 0.383). Hypothesis 5 was not supported. Supporting Hypothesis 6, employee self-care was negatively related to irritation (*γ* = −0.43, *p* < 0.001) and psychosomatic complaints (*γ* = −0.32, *p* < 0.001), and positively to overall health (*γ* = 0.33, *p* < 0.001). Hypothesis 7 postulated that leader self-care would relate to employee health via employee self-care (the role modeling hypothesis). Indirect effects of leader self-care on employee health via employee self-care were not significant (see Table 3). Hypothesis 7 was not supported. 

Due to missing values for some of the control variables (age: 3%; gender: 1%; span of control: 2%), one leader and 29 employees were excluded from the analyses, so that the effective sample size for the path models was N = 437 on Level 1 and N = 46 on Level 2. As a robustness check, we tested whether the model would also hold in the full sample without control variables. Only the indirect effect of leader self-care on overall health via leader staff care and employee staff care was reduced (*γ* = 0.06, SE = 0.03, *p* < 0.10; 95%-CI [0.01, 0.11]); all other relationships and significance levels remained the same.

## 4. Discussion

The aim of this study was to investigate how employees may benefit from self-caring leaders by testing two different explanations: (a) the improved leadership hypothesis, which states that leader self-care is related to employee health via improved staff care, and (b) the role-modeling hypothesis, stating that leader self-care relates to employee health via improved employee self-care. Combining leader self-care with leader and employee ratings of staff care, our findings advance insights into the role of leader resources for healthy leadership (see [8,9]) and add to a small number of multi-source studies of health-oriented leadership [17,29].

Consistent with the first set of hypotheses, we found that leaders with more self-care also tended to report higher staff care (H1), which related to higher employee staff care (H2). In turn, higher employee staff care was associated with lower irritation, fewer psychosomatic complaints, and better overall health (H3). We also found an indirect effect of leader self-care on irritation and overall health via leader staff care and employee staff care (H4). Additionally, all of the individual paths between the variables making up the serial indirect relationships were significant; thus, the findings are overall supportive of our theoretical considerations [71]. The pattern of relationships was consistent with the improved leadership hypothesis and underscores the relevance of self-care as a determinant of healthy leadership [10,17]. By comparison, the reported relationships were moderate, and previous research based on employee ratings suggests that self-care and staff care do not always go hand-in-hand [16]. The present study thus provides a useful starting point to explore moderators at the organizational, team, or individual level that can explain when leaders are more or less likely to translate their self-care into staff care.

In line with previous research [12,13,15], employee self-care was associated with lower strain and better health (H6). In contrast to our expectations, we found no support for the role-modeling hypothesis. Leader self-care did not directly relate to employee self-care (H5), nor indirectly to employee health via employee self-care (H7). This finding stands in contrast to research on absenteeism and presenteeism [4,56] or trickle-down models of leadership behavior [53]. First, there may be methodological reasons for this. Once staff care is accounted for, higher statistical power may be required to detect a remaining direct relationship between leader self-care and employee self-care. It is also possible that, in contrast to leaders’ presence or absence at work, self-care behavior (e.g., taking breaks, good time management) may not be as readily observable for employees. Another reason may be task related, simply because leaders and employees do not have the same job: with regard to self-care, employees without leadership responsibilities may find that what works for their leader may not be feasible or useful for them. Again, future research should explore moderators to identify when employees are more or less likely to adopt self-care behavior from their leader. 

### 4.1. Implications for Theory and Practice

The findings of this study support the Health-oriented Leadership model [10] by highlighting the relevance of leader self-care for healthy leadership and employee outcomes. We also provide initial evidence why leader self-care matters: leader self-care tends to go along with staff care, which in turn relates to employee health. This is in line with the notion in COR theory that people are more able and willing to expand resources for others when their own resources are secure [8,72]. The relationship we found was indirect via shared perceptions of staff care among leaders and employees, but there was no direct relationship between leader self-care and employee self-care. This suggests that self-care does not simply trickle down as employees emulate their leaders’ behavior. Instead, leader self-care seems to affect employees primarily to the extent that it is translated into staff care and that employees recognize staff care. The exact mechanisms still need to be further unpacked. Future studies could test and compare different theoretical pathways: For example, resource-based mechanisms (see [72]), motivational explanations stemming from a desire for consistency (see [47]), or self-regulation strategies required for both self-leadership and self-leadership (see [46]) may all potentially explain why leader self-care relates to staff care. Further theoretical and empirical work is also needed to explore when self-caring leaders show more staff care and how this is received by employees (see [35]). As the present study focused on behavior, other facets of health-oriented leadership (i.e., value and awareness) may play a moderating role, in addition to discrepancies between employees’ expectations and actual behavior [15]. With regard to role-modeling mechanisms, a closer application of social learning theory [22], taking into account employee expectancies or self-efficacy, may help clarify when and why employees adopt their leaders’ self-care behavior.

Several practical implications also follow from our findings. First, it seems vital that leaders are trained in self-care strategies, in addition to raising their awareness for their employees’ health at work. Interventions for leaders could aim at finding a good balance between self-care and staff care to prevent exhaustion through tending to others’ health [6]. Second, employees should be encouraged to actively maintain their health at work and supported with regard to their self-care; for example, by participating in occupational health promotion programs [40]. Finally, organizations should aim at creating a healthy work environment for both leaders and employees. If leaders are expected to tend to their employees’ health, they need to be equipped with the necessary resources to do so and maintain self-care at the same time.

### 4.2. Limitations and Avenues for Future Research

Some limitations of this study need to be addressed when discussing the results. First, the direction of effects is not clear from the cross-sectional nature of our study. Although data was collected at two points in time, we cannot rule out reversed causality. This is especially important to consider in mediation analysis, which implies a causal chain between variables. It is therefore important to keep in mind that we have established indirect relationships, not effects, between leader self-care, staff care, and employee well-being. It is, for example, plausible to assume that leaders’ self-care and staff care partly represent reactions to their employees and not just one-directional influences [55,73], or that employees’ health shapes their perception of staff care [2]. Previous longitudinal research has linked staff care to subsequent trajectories of physical and mental health over several months [14]. However, most employees in our sample had worked with their leaders for several years, so that reciprocal effects between leader behavior and employee health have likely already developed for quite some time, while the timeframe for the onset of effects is unknown. In such cases, a cross-sectional design is useful to establish a covariation between the variables of interest [51]. Nevertheless, we want to reiterate calls for more longitudinal studies that specifically address reciprocal relationships between leader self-care, staff care, and health over time, in addition to relevant timeframes for effects to develop [5].

A second limitation to causality relates to alternative explanations through the influence of third variables, which can never be controlled completely in any observational study [51]. Accordingly, we cannot rule out that part of the observed relationships between the variables of interest may be caused by unobserved structural or cultural differences between departments or individual differences among leaders and employees, which simultaneously influence self-care, staff care, and health outcomes. However, we did control for employee age, gender, and tenure with the leader. We also controlled for the span of control to ensure that associations between leader self-care and staff care were not simply a function of the demands associated with having to manage larger vs. smaller teams [65]. Future research would nevertheless benefit from including additional contextual factors, such as organizational climate or work design, and addressing substantive research questions about the role of contextual moderators in the relationship between the HoL concepts and health (see [26,35]). 

Despite the strengths of the multi-source design, we cannot rule out that common-method variance may have inflated relationships between variables within the groups of leaders and employees [74]. However, the consistent positive relationship between leader and employee ratings of staff care shows that the two rating sources share a common core. Regarding measurement, it should also be noted that the fit of our model was not optimal as some item loadings were low. Although there was one outlier item with a low loading, especially on the self-care scale, some of the comparably lower loadings for both self-care and staff care were observed for (a) specific items referring to occupational health promotion and safety rules at work, which are not always available or relevant, (b) reverse-coded items (health-risking behavior), and (c) items that refer to personal health behavior outside work (personal lifestyle). Although these facets belong to the behavioral subdimension, a differentiated analysis, even at a single item level, may be useful in a consulting context. In order to have a parsimonious model we focused on overall self-care and staff care behavior in our study. Combining these facets into one behavior scale is justified as profile analyses suggest that health-promoting and health-risking behavior tend to go hand-in-hand, i.e., when one is low, the other tends to be high and vice versa [16]. Altogether, our measurement model supports self-care and staff care as empirically distinct constructs, as does previous research [12,13,15]. Completely spurious correlations thus seem unlikely. Future studies may incorporate physiological health indicators to reduce common-method bias. 

Finally, we measured overall health status with a single item, which may limit validity and reliability. However, single item-measures for self-rated health have been studied extensively and have been shown to be valid and reliable measures of physical and mental well-being [19,20,75]. Furthermore, the item showed significant correlations with irritation and psychosomatic complaints, and the path model results give us little reason to suspect that the item performed worse than the multi-item scales. 

## 5. Conclusions

This study was the first to integrate leaders’ and employees’ perspectives on health-oriented leadership and investigate alternative mechanisms that may link leader self-care to employee health. The findings support the improved leadership hypothesis rather than direct role-modeling and underline self-care as a determinant of healthy leadership. Self-caring leaders were found to report more staff care than those low on self-care. Accordingly, their employees perceive higher staff care and report lower strain and better health. More research is needed to understand when and how leaders serve as role models such that employees adopt their self-care behavior. Organizations should equip leaders with the necessary resources to maintain a healthy balance between self-care and tending to employee health. 

## Figures and Tables

**Table 1:** Descriptive statistics and bivariate correlations between all study variables.

	M (SD)/%	ICC	1	2	3	4	5	6	7	8	9	10	11	12	13
Level 1: Employee variables															
**1** Age	44.67 (10.94)		–	−0.09	0.39 **	−0.02	−0.17	0.25	0.09	0.30 *	0.13	−0.24			
**2** Gender	0.78 (0.42)		0.03	–	−0.19	0.17	0.02	0.03	−0.17	0.18	0.45 **	−0.23			
**3** Tenure with leader	4.35 (4.37)		0.30 ***	−0.04	–	0.17	0.34 *	0.06	−0.06	0.14	−0.08	0.05			
**4** Employee self-care	3.19 (0.58)	0.07	0.06	0.06	0.07	(0.79)	0.53 ***	−0.48 **	−0.43 **	−0.38 **	−0.38 **	0.52 ***			
**5** Employee staff care	3.23 (0.81)	0.20	−0.05	−0.05	0.07	0.46 ***	(0.93)	−0.51 ***	−0.53 ***	−0.35 *	−0.28	0.31 *			
**6** Irritation global	2.34 (0.88)	0.05	0.10 *	0.04	0.01	−0.49 ***	−0.37 ***	(0.86)	0.81 ***	0.87 ***	0.43 **	−0.54 ***			
**7** Cognitive irritation	2.64 (1.18)	0.09	0.07	−0.05	−0.04	−0.45 ***	−0.31 ***	0.80 ***	(0.86)	0.42 **	0.14	−0.24			
**8** Emotional irritation	2.17 (0.94)	0.04	0.09 *	0.10 *	0.05	−0.39 ***	−0.32 ***	0.89 ***	0.44 ***	(0.85)	0.55 ***	−0.63 ***			
**9** Psychosomatic complaints	2.30 (0.85)	0.10	0.07	0.24 ***	−0.01	−0.36 ***	−0.28 ***	0.57 ***	0.43 ***	0.53 ***	(0.65)	−0.79 ***			
**10** Overall health	7.42 (1.75)	0.09	−0.19 ***	−0.08	0.00	0.40 ***	0.30 ***	−0.47 ***	−0.30 ***	−0.47 ***	−0.54 ***	–			
Level 2: Leader variables															
**11** Span of control	13.89 (5.92)		0.20	0.17	0.05	−0.08	−0.15	0.20	0.08	0.25	0.28	−0.32 *	–		
**12** Leader self-care	3.21 (0.61)		0.06	0.07	−0.03	0.05	0.24	−0.19	−0.19	−0.13	0.07	−0.03	0.30 *	(0.85)	
**13** Leader staff care	3.64 (0.49)		−0.07	0.22	−0.04	0.21	0.36*	−0.18	−0.18	−0.12	0.03	0.09	0.12	0.61 ***	(0.86)

*N* = 445–466 due to pairwise deletion of missing values on Level 1; *N* = 47 on Level 2. * *p* < 0.05, ** *p* < 0.01, *** *p* < 0.01. Correlations between employee and leader variables based on group means of the respective employee variables (employee gender was aggregated as percentage of women per team). Team-level correlations above the diagonal; Cronbach’s α in parentheses across the diagonal.

**Table 2:** Standardized coefficients from multilevel path analysis of irritation, psychosomatic complaints, and overall health.

	Irritation	Psychosomatic Complaints	Overall Health
*Level 1*	*γ* (SE)	*γ* (SE)	*γ* (SE)
**Employee staff care**			
Age	0.08 (0.05)	0.08 (0.05)	0.08 (0.05)
Gender	0.03 (0.05)	0.03 (0.05)	0.03 (0.05)
Tenure with leader	0.11 (0.06) †	0.11 (0.06) †	0.11 (0.06) †
**Employee self-care**			
Age	0.04 (0.05)	0.04 (0.05)	0.04 (0.05)
Gender	0.07 (0.04)	0.07 (0.04)	0.07 (0.04)
Tenure with leader	0.07 (0.05)	0.07 (0.05)	0.07 (0.05)
**Health outcomes**			
Age	0.12 (0.05) *	0.10 (0.05) *	−0.22 (0.05) ***
Gender	0.04 (0.04)	0.24 (0.04) ***	−0.07 (0.05)
Tenure with leader	0.01 (0.06)	−0.02 (0.04)	0.06 (0.06)
Employee staff care (b2.1)	−0.18 (0.05) **	−0.15 (0.07) *	0.18 (0.06) **
Employee self-care (b3.1)	−0.43 (0.04) ***	−0.32 (0.05) ***	0.33 (0.05) ***
*Level 2*	*γ* (SE)	*γ* (SE)	*γ* (SE)
**Leader staff care**			
Span of control	0.27 (0.10) **	0.27 (0.10) **	0.27 (0.10) **
Leader self-care (a1)	0.62 (0.07) ***	0.62 (0.07) ***	0.62 (0.07) ***
**Employee staff care**			
Span of control	−0.23 (0.10) *	−0.23 (0.10) *	−0.23 (0.10) *
Leader self-care (a2.2)	0.02 (0.18)	0.02 (0.18)	0.02 (0.18)
Leader staff care (d1)	0.47 (0.15) **	0.47 (0.15) **	0.47 (0.15) **
**Employee self-care**			
Span of control	0.11 (0.13)	0.11 (0.13)	0.11 (0.13)
Leader self-care (a3.2)	0.11 (0.13)	0.11 (0.13)	0.11 (0.13)
**Health outcomes**			
Span of control	0.37 (0.36)	−0.47 (0.17) **	−0.47 (0.16) **
Leader self-care (c’)	−0.49 (0.37)	0.11 (0.22)	−0.11 (0.21)
Leader staff care (b1)	0.02 (0.34)	−0.15 (0.21)	0.40 (0.21) †
Employee staff care (b2.2)	0.19 (0.49)	0.39 (0.24)	−0.75 (0.22) **
Employee self-care (b3.2)	0.02 (0.41)	−0.38 (0.23) †	0.53 (0.18) **
*Variance components*			
Residual variance Level 1	0.54	0.53	0.76
Residual variance Level 2	0.01	0.03	0.23
*R^2^* Level 1	0.31 ***	0.23 ***	0.24 ***

*N* = 437 on Level 1, *N* = 46 on Level 2. Correlations between employee staff care and employee self-care are not displayed but included in the model. † *p* < 0.10, * *p* < 0.05, ** *p* < 0.01, *** *p* < 0.001.

**Table 3:** Unstandardized indirect effects from multilevel path analysis of irritation, psychosomatic complaints, and overall health.

	Irritation	Psychosomatic Complaints	Overall Health
*Indirect effects*	*γ* (SE)	*γ* (SE)	*γ* (SE)
**Leader self-care** ** → Leader staff care (M1)** ** → Employee staff care (M2)** ** → Employee health**			
Within-indirect effect	−0.04 (0.02) *	−0.03 (0.02) †	0.08 (0.04) *
Between-indirect effect	0.01 (0.03)	0.04 (0.04)	−0.17 (0.10) †
Total effect via staff care	−0.14 (0.06) *	0.02 (0.07)	0.01 (0.13)
**Leader self-care** ** → Employee self-care** ** → Employee health**			
Within-indirect effect	−0.03 (0.03)	−0.02 (0.02)	0.04 (0.05)
Between-indirect effect	0.00 (0.01)	−0.02 (0.02)	0.05 (0.06)
Total effect via self-care	−0.14 (0.08) †	0.01 (0.10)	0.00 (0.18)

*N* = 437 on Level 1, *N* = 46 on Level 2. Indirect effects calculated from the path models presented in Table 2. Number of free parameters: 37. Correlations between employee staff care and employees self-care are not displayed but included in the model. † *p* < 0.10, * *p* < 0.05.

## Data Availability

The data are available from the authors upon reasonable request.

## Supplementary Materials

The following supporting information can be downloaded at: https://www.mdpi.com/article/10.3390/ijerph19116733/s1, Table S1: Overview of item loadings in the CFA on employee self care and staff care. Table S2: Overview of variable names and Mplus code for the cross-cluster mediation model.

## References

[1] Yang T., Shen Y.-M., Zhu M., Liu Y., Deng J., Chen Q., See L.-C., Yang T., Shen Y.-M., Zhu M. (2015). Effects of Co-Worker and Supervisor Support on Job Stress and Presenteeism in an Aging Workforce: A Structural Equation Modelling Approach. Int. J. Environ. Res. Public Health.

[2] Nielsen K., Randall R., Yarker J., Brenner S.-O. (2008). The Effects of Transformational Leadership on Followers’ Perceived Work Characteristics and Psychological Well-Being: A Longitudinal Study. Work Stress.

[3] Li Y., Wang Z., Yang L.-Q., Liu S. (2016). The Crossover of Psychological Distress from Leaders to Subordinates in Teams: The Role of Abusive Supervision, Psychological Capital, and Team Performance. J. Occup. Health Psychol..

[4] Dietz C., Zacher H., Scheel T., Otto K., Rigotti T. (2020). Leaders as Role Models: Effects of Leader Presenteeism on Employee Presenteeism and Sick Leave. Work Stress.

[5] Nielsen K., Taris T.W. (2019). Leading Well: Challenges to Researching Leadership in Occupational Health Psychology–and Some Ways Forward. Work Stress.

[6] Barling J., Cloutier A. (2017). Leaders’ Mental Health at Work: Empirical, Methodological, and Policy Directions. J. Occup. Health Psychol..

[7] Kaluza A.J., Boer D., Buengeler C., van Dick R. (2020). Leadership Behaviour and Leader Self-Reported Well-Being: A Review, Integration and Meta-Analytic Examination. Work Stress.

[8] Tafvelin S., Nielsen K., von Thiele Schwarz U., Stenling A. (2019). Leading Well Is a Matter of Resources: Leader Vigour and Peer Support Augments the Relationship between Transformational Leadership and Burnout. Work Stress.

[9] Fladerer M.P., Braun S. (2020). Managers’ Resources for Authentic Leadership—A Multi-Study Exploration of Positive Psychological Capacities and Ethical Organizational Climates. Br. J. Manag..

[10] Franke F., Felfe J., Pundt A. (2014). The Impact of Health-Oriented Leadership on Follower Health: Development and Test of a New Instrument Measuring Health-Promoting Leadership. Ger. J. Hum. Resour. Manag..

[11] Inceoglu I., Thomas G., Chu C., Plans D., Gerbasi A. (2018). Leadership Behavior and Employee Well-Being: An Integrated Review and a Future Research Agenda. Leadersh. Q..

[12] Horstmann D. (2018). Enhancing Employee Self-Care: The Moderating Effect of Personal Initiative on Health-Specific Leadership. Eur. J. Health Psychol..

[13] Santa Maria A., Wolter C., Gusy B., Kleiber D., Renneberg B. (2019). The Impact of Health-Oriented Leadership on Police Officers’ Physical Health, Burnout, Depression and Well-Being. Polic. A J. Policy Pract..

[14] Arnold M., Rigotti T. (2020). Is It Getting Better or Worse? Health-Oriented Leadership and Psychological Capital as Resources for Sustained Health in Newcomers. Appl. Psychol. An Int. Rev..

[15] Kaluza A.J., Weber F., van Dick R., Junker N.M. (2021). When and How Health-Oriented Leadership Relates to Employee Well-Being—The Role of Expectations, Self-Care, and LMX. J. Appl. Soc. Psychol..

[16] Klug K., Felfe J., Krick A. (2019). Caring for Oneself or for Others? How Consistent and Inconsistent Profiles of Health-Oriented Leadership Are Related to Follower Strain and Health. Front. Psychol..

[17] Grimm L.A., Bauer G.F., Jenny G.J. (2021). Is the Health-Awareness of Leaders Related to the Working Conditions, Engagement, and Exhaustion in Their Teams? A Multi-Level Mediation Study. BMC Public Health.

[18] Sonnentag S., Frese M., Borman W.C., Ilgen D.R., Klimoski R. (2002). Stress in Organizations. Comprehensive Handbook of Psychology: Industrial and organizational Psychology.

[19] Singh-Manoux A., Martikainen P., Ferrie J., Zins M., Marmot M., Goldberg M. (2006). What Does Self Rated Health Measure? Results from the British Whitehall II and French Gazel Cohort Studies. J. Epidemiol. Community Health.

[20] Baćak V., Ólafsdóttir S. (2017). Gender and Validity of Self-Rated Health in Nineteen European Countries. Scand. J. Public Health.

[21] Hobfoll S.E. (1989). Conservation of Resources: A New Attempt at Conceptualizing Stress. Am. Psychol..

[22] Bandura A. (1971). Social Learning Theory.

[23] Montano D., Reeske A., Franke F., Hüffmeier J. (2017). Leadership, Followers’ Mental Health and Job Performance in Organizations: A Comprehensive Meta-Analysis from an Occupational Health Perspective. J. Organ. Behav..

[24] Harms P.D., Credé M., Tynan M., Leon M., Jeung W. (2017). Leadership and Stress: A Meta-Analytic Review. Leadersh. Q..

[25] Schyns B., Schilling J. (2013). How Bad Are the Effects of Bad Leaders? A Meta-Analysis of Destructive Leadership and Its Outcomes. Leadersh. Q..

[26] Rudolph C.W., Murphy L.D., Zacher H. (2020). A Systematic Review and Critique of Research on “Healthy Leadership”. Leadersh. Q..

[27] Vincent-Höper S., Stein M. (2019). The Role of Leaders in Designing Employees’ Work Characteristics: Validation of the Health- and Development-Promoting Leadership Behavior Questionnaire. Front. Psychol..

[28] Gurt J., Schwennen C., Elke G. (2011). Health-Specific Leadership: Is There an Association between Leader Consideration for the Health of Employees and Their Strain and Well-Being?. Work Stress.

[29] Vonderlin R., Schmidt B., Müller G., Biermann M., Kleindienst N., Bohus M., Lyssenko L. (2021). Health-Oriented Leadership and Mental Health From Supervisor and Employee Perspectives: A Multilevel and Multisource Approach. Front. Psychol..

[30] Richards K.C., Campenni C.E., Muse-Burke J.L. (2010). Self-Care and Well-Being in Mental Health Professionals: The Mediating Effects of Self-Awareness and Mindfulness. J. Ment. Health Couns..

[31] Cook-Cottone C.P., Guyker W.M. (2018). The Development and Validation of the Mindful Self-Care Scale (MSCS): An Assessment of Practices That Support Positive Embodiment. Mindfulness.

[32] Levin L.S., Idler E.L. (1983). Self-Care in Health. Annu. Rev. Public Health.

[33] McGarrigle T., Walsh C.A. (2011). Mindfulness, Self-Care, and Wellness in Social Work: Effects of Contemplative Training. J. Relig. Spiritual. Soc. Work.

[34] Rudaz M., Twohig M.P., Ong C.W., Levin M.E. (2017). Mindfulness and Acceptance-Based Trainings for Fostering Self-Care and Reducing Stress in Mental Health Professionals: A Systematic Review. J. Context. Behav. Sci..

[35] Klebe L., Felfe J., Klug K. (2021). Healthy Leadership in Turbulent Times: The Effectiveness of Health-Oriented Leadership in Crisis. Br. J. Manag..

[36] Horstmann D., Remdisch S. (2016). Gesundheitsorientierte Führung in Der Altenpflege. Zeitschrift für Arbeits-und Organisationspsychologie A&O.

[37] Köppe C., Kammerhoff J., Schütz A. (2018). Leader-Follower Crossover: Exhaustion Predicts Somatic Complaints via StaffCare Behavior. J. Manag. Psychol..

[38] Krick A., Felfe J., Pischel S. (2022). Health-Oriented Leadership as a Job Resource: Can Staff Care Buffer the Effects of Job Demands on Employee Health and Job Satisfaction?. J. Manag. Psychol..

[39] Klebe L., Klug K., Felfe J. (2021). The Show Must Go On. Zeitschrift für Arbeits-und Organisationspsychologie A&O.

[40] Krick A., Felfe J., Klug K. (2019). Turning Intention into Participation in Occupational Health Promotion Courses? The Moderating Role of Organizational, Intrapersonal, and Interpersonal Factors. J. Occup. Environ. Med..

[41] Krick A., Felfe J., Klug K. (2021). Building Resilience: Trajectories of Heart Rate Variability during a Mindfulness-Based Intervention and the Role of Individual and Social Characteristics. Int. J. Stress Manag..

[42] Cavanaugh M.A., Boswell W.R., Roehling M.V., Boudreau J.W. (2000). An Empirical Examination of Self-Reported Work Stress among U.S. Managers. J. Appl. Psychol..

[43] Knudsen H.K., Ducharme L.J., Roman P.M. (2009). Turnover Intention and Emotional Exhaustion “at the Top”: Adapting the Job Demands-Resources Model to Leaders of Addiction Treatment Organizations. J. Occup. Health Psychol..

[44] Neck C.P., Houghton J.D. (2006). Two Decades of Self-Leadership Theory and Research: Past Developments, Present Trends, and Future Possibilities. J. Manag. Psychol..

[45] Manz C.C., Sims H.P. (1991). Super Leadership: Beyond the Myth of Heroic Leadership. Organ. Dyn..

[46] Furtner M.R., Baldegger U., Rauthmann J.F. (2013). Leading Yourself and Leading Others: Linking Self-Leadership to Transformational, Transactional, and Laissez-Faire Leadership. Eur. J. Work Organ. Psychol..

[47] Mayer D.M., Aquino K., Greenbaum R.L., Kuenzi M. (2012). Who Displays Ethical Leadership, and Why Does It Matter? An Examination of Antecedents and Consequences of Ethical Leadership. Acad. Manag. J..

[48] Stein M., Vincent-Höper S., Gregersen S. (2020). Why Busy Leaders May Have Exhausted Followers: A Multilevel Perspective on Supportive Leadership. Leadersh. Organ. Dev. J..

[49] Lee A., Carpenter N.C. (2018). Seeing Eye to Eye: A Meta-Analysis of Self-Other Agreement of Leadership. Leadersh. Q..

[50] Ostroff C., Atwater L.E., Feinberg B.J. (2004). Understanding Self-Other Agreement: A Look at Rater and Ratee Characteristics, Context and Outcomes. Pers. Psychol..

[51] Spector P.E. (2019). Do Not Cross Me: Optimizing the Use of Cross-Sectional Designs. J. Bus. Psychol..

[52] Kelloway E.K., Barling J. (2010). Leadership Development as an Intervention in Occupational Health Psychology. Work Stress.

[53] Wo D.X.H., Schminke M., Ambrose M.L. (2019). Trickle-down, Trickle-out, Trickle-up, Trickle-in, and Trickle-around Effects: An Integrative Perspective on Indirect Social Influence Phenomena. J. Manag..

[54] Westman M. (2001). Stress and Strain Crossover. Hum. Relations.

[55] Wirtz N., Rigotti T., Otto K., Loeb C. (2017). What about the Leader? Crossover of Emotional Exhaustion and Work Engagement from Followers to Leaders. J. Occup. Health Psychol..

[56] Løkke Nielsen A.-K. (2008). Determinants of Absenteeismin Public Organizations: A Unit-Level Analysis of Work Absence in a Large Danish Municipality. Int. J. Hum. Resour. Manag..

[57] Pundt F., Felfe J. (2017). Health Oriented Leadership. Instrument Zur Erfassung Gesundheitsförderlicher Führung.

[58] Mohr G., Müller A., Rigotti T., Aycan Z., Tschan F. (2006). The Assessment of Psychological Strain in Work Contexts. Eur. J. Psychol. Assess..

[59] Mohr G., Rigotti T., Müller A. (2009). Irritation Scale for the Assessment of Work-Related Strain.

[60] Mohr G. (1986). Die Erfassung Psychischer Befindensbeeinträchtigungen Bei Industriearbeitern.

[61] Nübling M., Stößel U., Hasselhorn H.-M., Michaelis M., Hofmann F. (2006). Measuring Psychological Stress and Strain at Work—Evaluation of the COPSOQ Questionnaire in Germany. GMS Psycho-Social Med..

[62] Macintyre S., Hunt K., Sweeting H. (1996). Gender Differences in Health: Are Things Really as Simple as They Seem?. Soc. Sci. Med..

[63] Marmot M., Allen J., Bell R., Bloomer E., Goldblatt P. (2012). WHO European Review of Social Determinants of Health and the Health Divide. Lancet.

[64] Sin H.-P., Nahrgang J.D., Morgeson F.P. (2009). Understanding Why They Don’t See Eye to Eye: An Examination of Leader–Member Exchange (LMX) Agreement. J. Appl. Psychol..

[65] Thiel C.E., Hardy J.H., Peterson D.R., Welsh D.T., Bonner J.M. (2018). Too Many Sheep in the Flock? Span of Control Attenuates the Influence of Ethical Leadership. J. Appl. Psychol..

[66] Muthén L.K., Muthén B.O. (1998–2017). Mplus User’s Guide.

[67] Pituch K.A., Stapleton L.M. (2012). Distinguishing between Cross-and Cluster-Level Mediation Processes in the Cluster Randomized Trial. Sociol. Methods Res..

[68] Preacher K.J., Zyphur M.J., Zhang Z. (2010). A General Multilevel SEM Framework for Assessing Multilevel Mediation. Psychol. Methods.

[69] Stride C.B., Gardner S., Catley N., Thomas F. Mplus Code for Mediation, Moderation, and Moderated Mediation Models. http://www.offbeat.group.shef.ac.uk/FIO/mplusmedmod.htm.

[70] Hayes A.F. (2017). Introduction to Mediation, Moderation, and Conditional Process Analysis.

[71] Rucker D.D., Preacher K.J., Tormala Z.L., Petty R.E. (2011). Mediation Analysis in Social Psychology: Current Practices and New Recommendations. Soc. Personal. Psychol. Compass.

[72] Hobfoll S.E., Halbesleben J., Neveu J.-P., Westman M. (2018). Conservation of Resources in the Organizational Context: The Reality of Resources and Their Consequences. Annu. Rev. Organ. Psychol. Organ. Behav..

[73] Klebe L., Felfe J., Klug K. (2021). Mission Impossible? Effects of Crisis, Leader and Follower Strain on Health-Oriented Leadership. Eur. Manag. J..

[74] Podsakoff P.M., MacKenzie S.B., Lee J.-Y., Podsakoff N.P. (2003). Common Method Biases in Behavioral Research: A Critical Review of the Literature and Recommended Remedies. J. Appl. Psychol..

[75] Idler E.L., Benyamini Y. (1997). Self-Rated Health and Mortality: A Review of Twenty-Seven Community Studies. J. Health Soc. Behav..

