# Supplementary material for: Does Self-Care Make You a Better Leader? A Multisource Study Linking Leader Self-Care to Health-Oriented Leadership, Employee Self-Care, and Health
Source: Int J Environ Res Public Health. 2022 May 31;19(11):6733. doi: 10.3390/ijerph19116733 (PMC9180678; doi:10.3390/ijerph19116733)
Supplement: Supplementary file 1 [file ijerph-19-06733-s001.zip › ijerph-1701787-SI.pdf]

Table S1. Overview of item loadings in the CFA on employee self care and staff care

Item no.	Content	Factor loading
Self care		
Hol_sb1	I pay attention to a healthy lifestyle (e.g., healthy food, no smoking, doing sports)	.34
Hol_sb2	I do a lot for my health in my spare time	.39
Hol_sb3	I see to it that I get enough relaxation and recovery	.66
Hol_sb4	In my job it happens again and again that I pay too little attention to my health	.39
Hol_sb5*	When things have been stressful for a longer amount of time, I make sure to slow down afterwards	.72
Hol_sb6*	Oftentimes I expect too much of myself at work	.36
Hol_sb7	I regularly keep myself informed about workplace safety rules and offers for worksite health promotion	.32
Hol_sb8	Even in face of high time pressure, I consistently follow the safety rules	.40
Hol_sb9*	If there is a lot of work to do I sometimes skip my breaks	.32
Hol_sb10	I take advantage of workplace health promotion offers (e.g., back exercises, relaxation, stress management)	.14
Hol_sb11	I try to reduce my demands by optimizing my personal work routine (e.g., set priorities, care for undisturbed working, daily planning)	.59
Hol_sb12	I try to reduce my demands by optimizing my working conditions (e.g., avoid unbalanced body-posture, care for enough, space, avoid draft)	.54
Hol_sb13	I try to reduce my demands by optimizing my personal work-life balance (e.g., take regular breaks, avoid overtime, no work on the weekend, avoid the expiration of vacation days)	.61
Hol_sb14	I see to it that I have enough support when I need it	.62
Staff care		
Hol_mb1	My supervisor encourages me to care for a healthy lifestyle (e.g., healthy food, no smoking, doing sports)	.65
Hol_mb2	My supervisor regularly motivates me to do a lot for health in my spare time	.64
Hol_mb3	My supervisor tries to be a good role model for me with regard to health	.67
Hol_mb4	My supervisor sees to it that I have enough relaxation and recovery	.77
Hol_mb5*	In my job it often happens that my supervisor pays too little attention to my health	.46
Hol_mb6	When work has been stressful for a longer period of time, my supervisor ensures that things slow again afterwards	.66
Hol_mb7*	Oftentimes my supervisor expects too much of me	.48
Hol_mb8	My supervisor invites me to inform him/her about my work-related health risks	.66

Hol_mb9	My supervisor regularly keeps us informed about safety rules and worksite health promotion activities	.53
Hol_mb10	Even in face of high time pressure, my supervisor motivates me to consistently follow the safety rules	.66
Hol_mb11	When I seem to be stressed, my supervisor responds to it and tries to propose solutions	.70
Hol_mb12	When my supervisor is under the impression that someone is ill, he/she sends this person to the doctor or home	.62
Hol_mb13	My supervisor makes sure that the topic of health receives sufficient attention in our team	.80
Hol_mb14*	If there is a lot of work that needs to be done, then rest breaks sometimes have to be skipped	.24
Hol_mb15	My supervisor motivates me to take advantage of worksite health promotion offers (e.g., back exercises, relaxation, stress management)	.66
Hol_mb16	My supervisor tries to reduce my work demands by optimizing my work routines (e.g., set priorities, ensure for undisturbed work, daily planning)	.70
Hol_mb17	My supervisor tries to reduce my demands by optimizing my working conditions (e.g., avoid unbalanced body-posture, care for enough space, avoid draft)	.67
Hol_mb18	My supervisor tries to reduce my demands by optimizing my work-life balance (e.g., take regular breaks, avoid overtime, avoid the expiration of vacation days)	.75
Hol_mb19	By fostering positive interactions with one another, my supervisor sees to it that my work demands are reduced	.64

* reverse-coded item

Table S2. Overview of variable names and Mplus code for the cross-cluster mediation model

**Measures**	**Name in the Mplus code**
Team identifier	teamid
Age	age
Gender	sex
Tenure with leader	tenfk
Span of control	fkspan
Leader self care	fksb
Leader staff care	fkob
Employee staff care	mb
Employee self care	sb
Employee staff care aggregated to the team level	mb_m
Employee self care aggregated to the team level	sb_m
Irritation	irr

Mplus code for the cross-cluster mediation model (example for irritation as health outcome):

```
CLUSTER = teamid;
BETWEEN = fkspan fksb fkob mb_m sb_m;
WITHIN = age sex tenfk mb sb;
DEFINE:
  mb_m = CLUSTER_MEAN(mb);
  sb_m = CLUSTER_MEAN(sb);
  CENTER age tenfk fkspan sb mb fksb fkob mb_m sb_m(GRANDMEAN);
ANALYSIS:
  TYPE = TWOLEVEL;
  ESTIMATOR = MLR;
MODEL:
%WITHIN%
  !controls
  irr sb mb ON age sex tenfk;

  !within-team direct effects of employee staff care on health
  irr ON mb(b2_w);

  !within-team direct effects of employee self care on health
  irr ON sb(b12_w);

  !within-team effect of employee staff care on self care
  sb WITH mb;

%BETWEEN%
  !controls
  irr sb_m mb_m fkob ON fkspan;
```

```
  !direct effect of leader self care on employee self care (a1-path)
  sb_m ON fksb(a12);

  !direct effect of leader self care on leader staff care (a1-path)
  fkob ON fksb(a1);

  !direct effect of leader self care on employee staff care (a2-path)
  mb_m ON fksb(a2);

  !direct effect of staff care on health
  irr ON fkob(b1);

  !direct effect of staff care on health
  irr ON mb_m(b2_b);

  !direct effect of self care on health
  irr ON sb_m(b12_b);

  !direct effect of leader staff care on employee staff care
  mb_m ON fkob(d1);

  !effect of leader staff care on health
  irr ON fksb(c);

MODEL CONSTRAINT:
NEW(a1b1 a2b2_w a2b2_b a1d1b2_w a1d1b2_b TOTSTAFF a12b12_w a12b12_b
TOTSELF);

a1b1 = a1*b1; !Specific indirect effect of leader self care on health via leader staff care
a2b2_w = a2*b2_w; !Specific within-indirect effect of leader self care on health via employee
staff care
a2b2_b = a2*b2_b; !Specific between-indirect effect of leader self care on health via
employee staff care
a1d1b2_w = a1*d1*b2_w; !Specific within-indirect effect of leader self care on health via
leader and employee staff care
a1d1b2_b = a1*d1*b2_b; !Specific between-indirect effect of leader self care on health via
leader and employee staff car
TOTSTAFF = c +a1b1 + a2b2_w + a2b2_b + a1d1b2_w + a1d1b2_b; !Total effect via staff
care

a12b12_w = a12*b12_w; !Indirect within-effect of leader self care on health via employee
self care
a12b12_b = a12*b12_b; !Indirect between-effect of leader self care on health via employee
self care
TOTSELF = c + a12*b12_w + a12*b12_b; !Total effect self care
```
